# Co-expression network analysis reveals PbTGA4 and PbAPRR2 as core transcription factors of drought response in an important timber species *Phoebe bournei*

**DOI:** 10.3389/fpls.2023.1297235

**Published:** 2024-01-08

**Authors:** Jinjian Yu, Ke Yin, Yan Liu, Yuhui Li, Junhong Zhang, Xiao Han, Zaikang Tong

**Affiliations:** State Key Laboratory of Subtropical Silviculture, College of Forestry and Biotechnology, Zhejiang A&F University, Hangzhou, China

**Keywords:** *Phoebe bournei*, drought stress, abscisic acid, transcriptome, transcription factor, co-expression network

## Abstract

*Phoebe bournei* is one of the main afforestation tree species in subtropical regions of China and is famous for its timber. Its distribution and growth are significantly impaired by water conditions. Thus, it is essential to understand the mechanism of the stress response in *P. bournei*. Here, we analyzed the phenotypic changes and transcriptomic rearrangement in the leaves and roots of *P. bournei* seedlings grown for 0 h, 1 h, 24 h, and 72 h under simulated drought conditions (10% PEG 6000). The results showed that drought stress inhibited plant photosynthesis and increased oxidoreductase activity and abscisic acid (ABA) accumulation. Spatio-temporal transcriptomic analysis identified 2836 and 3704 differentially expressed genes (DEGs) in leaves and roots, respectively. The responsive genes in different organs presented various expression profiles at different times. Gene co-expression network analysis identified two core transcription factors, *TGA4* and *APRR2*, from two modules that showed a strong positive correlation with ABA accumulation. Our study investigated the different responses of aboveground and belowground organs of *P. bournei* to drought stress and provides critical information for improving the drought resistance of this timber species.

## Introduction

Woody plants are an abundant source of lignocellulosic biomass for bioenergy production (An et al., 2021). Trees, as sessile organisms, need to cope with constantly changing environments (Wang et al., 2017). Drought stress is one of the major environmental factors influencing the geographical distribution and biomass accumulation of trees (Zhu, 2016). The frequency and intensity of drought will continue to increase (Gupta et al., 2020). When water is insufficient, water from different tissues of the plant converges toward mature tissues, resulting in a lack of water in young tissues and retarded growth (Wakamori and Mineno, 2019). To reduce water loss under drought stress, plants reduce stomatal opening and transpiration rates, which restrict the entry of carbon dioxide into the leaves and inhibit photosynthesis to impair biomass accumulation (Zhang et al., 2009; Li et al., 2018). At the same time, the reduced transpiration rate also leads to the obstruction of mineral transport in plants, which further aggravates physiological and metabolic disorders in plant cells, leading to the destruction of the cell membrane structure and impaired cell growth (Lu et al., 2018; Lin et al., 2022). Cell elongation is most sensitive to drought, and a slight water deficit can cause a significant decrease in the growth rate, resulting in dwarf plants (Marmagne et al., 2020).

Roots and leaves are the most critical organs regulating the water balance between soil and plants (Li et al., 2021). Roots regulate water and nutrient uptake and are the first to sense water deficits (Hein et al., 2016). When plants experience drought conditions, a decrease in water transport from the roots to the aboveground parts through the xylem directly signals drought stress to the plant (Mansinhos et al., 2022). Roots can be traced to coordinated cell division, elongation, and differentiation events to enhance their ability to absorb water (Rellan-Alvarez et al., 2016). Drought also induces plant roots to produce small peptides and transmit them through the vascular system to leaves, driving the synthesis of ABA in leaves (Christmann and Grill, 2018). Leaves are the primary organs for transpiration, which is the primary mechanism of water loss and is monitored by stomates (Rodrigues et al., 2019). The regulation of stomatal movement by ABA is an important way for plants to regulate water balance under drought stress (Li et al., 2021). Moreover, inhibited photosynthesis and induced ABA in plants under drought stress will accumulate a large amount of reactive oxygen species (ROS) in cells, resulting in cell damage (Zhang et al., 2021). Plants defend against excessive ROS damage to cells by increasing the activity of antioxidant enzymes such as peroxidase (POD) and superoxide dismutase (SOD) (Wu et al., 2022).

ABA is a classic plant stress-related hormone, and at least two ABA-dependent signaling pathways are involved in response to water stress (Valliyodan and Nguyen, 2006; Guo et al., 2021). Previous research has shown that members of the MYB, MYC, bZIP, and B3 families are directly induced to express ABA (Yang et al., 2019). These genes compose a complicated regulatory network for the conduction of ABA signals. Transcriptomic sequencing provides technical support for the elucidation of this complex biological process. This approach is used to understand the expression response of genes under stress at the whole genome level. Transcriptomic sequencing is significant for constructing the stress-responsive transcriptional regulatory network (Wang et al., 2014). Based on this approach, we found that several genes encoding bHLH, WRKY, NAC, DREB, SnRK, protein phosphatases, protein kinases, and protein-related enzymes involved in phosphatidylinositol synthesis and metabolism are involved in the drought stress response (Sakuraba et al., 2015; Vaziriyeganeh et al., 2021). The elucidation of these genes and related regulatory networks contributes to breeding drought-resistant plant genotypes.

*Phoebe bournei* (Hemsl.) Yang is an important commercial and ornamental tree endemic to southern China. Its wood is regarded as “noble wood” due to its high corrosion resistance, special scent, and visible golden-tinted texture (Zhang J. H. et al., 2018). A recent survey of species distribution patterns showed that *P. bournei* is mainly distributed in the subtropics, with an annual average temperature of 15°C to 20°C and yearly precipitation of approximately 1505 mm (Ge et al., 2014). Frequent extreme climate conditions and water scarcity have reduced the suitable areas for *P. bournei*, making afforestation difficult (Li et al., 2022). Thus, it is essential to understand the regulatory mechanism of the stress response in *P. bournei*. However, current research on drought stress in *P. bournei* has focused on the patterns of changes in biomass, photosynthesis, and antioxidant enzyme activity (Ge et al., 2014; Tang et al., 2020). Little is known about the coordination of the belowground and aboveground parts of *P. bournei* trees and the corresponding molecular mechanisms involved in the stress response. In this study, changes in photosynthesis, antioxidant enzyme activity, ABA content, and gene expression profiling in the leaves and roots of *P. bournei* under different treatment periods of drought stress were systematically analyzed from a temporal and an organ-level perspective. Gene co-expression analysis was employed to identify hub genes and key modules in *P. bournei* in response to drought stress. This study may inform future studies on the potential mechanisms underlying *P. bournei* drought resistance and provide theoretical support for genetic engineering to breed drought-resistant *P. bournei* varieties.

## Materials and methods

### Plant material and PEG treatment

Seedlings of *P. bournei* cv. ‘WY8’ were collected from the plantation base at Zhejiang Agriculture and Forestry University in Zhejiang Province in China (30°15′N, 119°43′E). Plants with uniform growth were selected and hydroponically cultured in ¼-strength Hoagland’s solution. Seedlings were cultured in a greenhouse at 25°C, 16 h/8 h (light/dark), and 60% humidity. The culture solution was renewed every four days. After one month of preculture, plants that showed consistent and vigorous growth were chosen for further experiments. PEG treatment was imposed on the plants by transplanting them into ¼-strength Hoagland’s solution containing 10% PEG 6000 for 0 h, 1 h, 24 h, and 72 h. To avoid the effects of the exploitation process on the experimental results, the experiment end time and the sampling period were standardized (Yang and Wei, 2015). Leaves and roots were collected from each group of ten individual plants and stored at -80 °C for physiological tests and RNA-Seq analysis.

### Measurements of the physiological indices of *P. bournei*

In this study, the photosynthetic parameters of the seedlings were monitored using a Li-6400 Portable Photosynthesis System (LI-COR Biosciences, Lincoln, NE, USA), with a controlled reference CO_2_ concentration of 400 μmol·mol^-1^ in the leaf chamber and photosynthetically active radiation set to 1200 μmol·mol^-1^·s^-1^. The measurements were carried out from 9:30 a.m. to 11:30 a.m. The photosynthetic parameters of 3–5 healthy and mature leaves of four groups of seedlings were automatically recorded by the experimental apparatus. The photosynthetic parameters included the net photosynthetic rate, transpiration rate, and stomatal conductance. Concurrently, the maximum photochemical efficiency (Fv/FM) of PSII after 30 minutes of dark adaptation was measured by a portable modulated chlorophyll fluorometer (PAM-2500, Heinz Walz GmbH, Germany). Superoxide dismutase (SOD) and peroxidase (POD) activities were determined with the corresponding kits (Nanjing Jincheng Bioengineering Institute, Nanjing, China). The ABA content was measured by MetWare (http://www.metware.cn/) based on the AB Sciex QTRAP 6500 LC-MS/MS platform (AB SCIEX, Foster City, CA, USA). Three replicates of each assay were performed. In this research, each physiological parameter was measured in triplicate.

### RNA extraction, library preparation, and mRNA sequencing

Total RNA was extracted from 200 mg of ground leaf or root tissue with the M5 HiPer Plant RNeasy Mini Kit (Mei5bio, Beijing, China) following the manufacturer’s protocol, with each tissue being analyzed in triplicate. The quantity and purity of RNA were determined with a NanoDrop 2000 (Thermo Fisher Scientific, New York, USA) and 1% agarose gel electrophoresis. The qualified RNA was used for library construction and transcriptomic sequencing. Eight treatments, L_0h (leaves of plants treated with 10% PEG for 0 h), L_1h (leaves of plants treated with 10% PEG for 1 h), L_24h (leaves of plants treated with 10% PEG for 24 h), L_72h (leaves of plants treated with 10% PEG for 72 h), R_0h (roots of plants treated with 10% PEG for 0 h), R_1h (roots of plants treated with 10% PEG for 1 h), R_24h (roots of plants treated with 10% PEG for 24 h), R_72h (roots of plants treated with 10% PEG for 72 h), totaling twenty-four cDNA libraries were constructed according to the manufacturer’s instructions (Illumina, Inc., San Diego, CA, USA). Using oligo dT25 magnetic beads, 10 μg of total RNA was converted into mRNA, which was subsequently fragmented into small fragments. With random hexamer primers, these fragments were used to create first-strand complementary DNA (cDNA), followed by second-strand cDNA using a dUTP mixture. Magnetic beads were used to separate the double-stranded cDNA, then treated with three single adenylations and end repair. Following size selection using the adenylated fragments, sequencing adaptors were ligated to them and then enriched by PCR amplification. During the quality control phase, an Agilent 2100 Bioanalyzer was used to evaluate the quantity and quality of the sample library. Following the vendor’s suggested methodology, paired-end 150-bp sequencing on an Illumina HiSeq2000 was carried out at LC Sciences (Houston, TX, USA). Transcriptomic sequencing was commissioned by LC-Bio Technologies Co., Ltd. (Hangzhou, China). Sequencing data have been uploaded to the National Genomics Data Center (NGDC)(Chen et al., 2021; Xue et al., 2022), and the assigned accession of the submission is CRA011591.

### Sequence mapping and annotation and differentially expressed gene analysis

After the adapter sequences were trimmed and the low-quality reads were filtered, the valid data were mapped onto the *P. bournei* genome (Han et al., 2022) by Tophat2 v2.0.6. The transcript abundance estimation was processed by Cufflinks v2.0.2. Genomic locations and UMI tags were used to remove redundancy and perform calibration to obtain comprehensive transcript information. We utilized protein sequence similarity, the Kyoto Encyclopedia of Genes and Genomes (KEGG), cluster of orthologous groups of proteins (COG), and gene ontology (GO) analyses to perform the functional annotation of unigenes. We aligned all paired-end reads back to the final assembly using Perl scripts in Trinity to investigate the expression level of each unigene in different samples and expressed the abundance of unigenes in transcripts per million (TPM). The differentially expressed genes (DEGs) were screened based on |log2(fold change)|>1 and *P*<0.05. The GO term enrichment analysis of the DEGs was carried out using the GOseq R package and plotted using the GOplot R package. PlantTFDB (http://planttfdb.gao-lab.org/) was used to analyze transcription factors (TFs) in DEGs.

### Weighted gene co-expression network analysis

The co-expression network and the correlation of modules and physiological data were analyzed by the R package WGCNA according to a previously described method (Langfelder and Horvath, 2008). The co-expression network was generated using genes with TPM > 1. We analyzed the correlation of module traits by combining the co-expression network, antioxidant enzyme activity, and ABA content. Key modules were screened through the correlation between modules and phenotypes. We selected the hub gene according to the module membership and gene significance of each gene in the key module. Cytoscape v3.7.2 was used to plot the co-expression network between the core TFs and co-expressed genes.

### qRT-PCR validation

The genes of interest were validated by qRT-PCR. Primer3 software (http://frodo.wi.mit.edu/primer3/input.htm) was used to design primers with an output production of 150–300 bp (**Supplementary Table 1**). *PbUBQ4* and *PbEF1α* were used as internal controls. We utilized the SYBR Premix EX Taq Kit (TaKaRa, Dalian, China) for qRT-PCR amplification on the CFX96 Real-Time PCR Detection System (Bio-Rad Laboratories, Hercules, CA, USA). The 2^-△△Ct^ method was used to calculate the relative expression of the target gene, and qRT-PCR analysis was conducted with three replicates of each cDNA sample. The resulting data are presented as the average value and standard deviation.

### Statistical analyses

SPSS22.0 (SPSS Inc., Chicago, IL, USA) was used for the statistical analysis of both physiological data and relative expression, with the significance of the difference between the data tested using One-way ANOVA and Duncan’s multiple comparisons (*P*<0.05).

## Results

### Morphological and physiological evaluation of *P. bournei* in response to PEG

**Figure 1:**
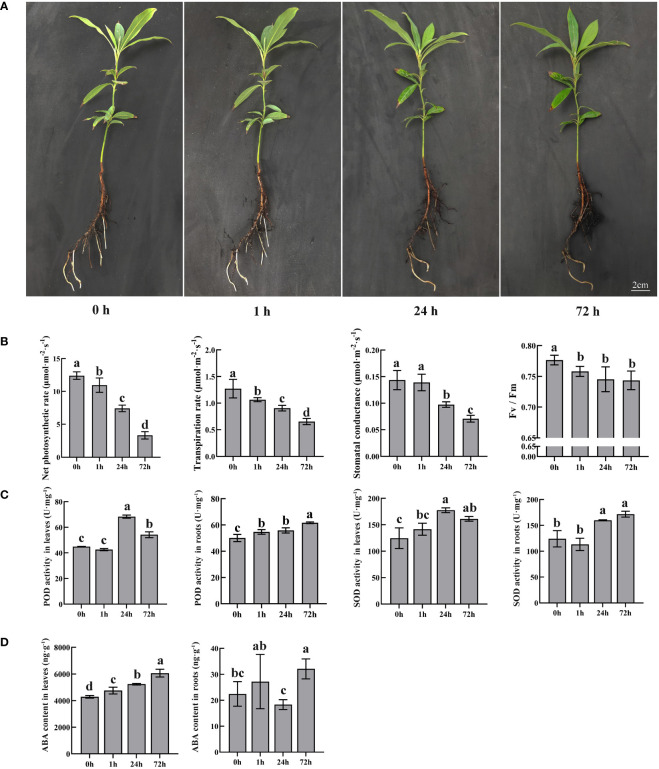
Morphological and physiological changes in *P. bournei* in response to PEG. **(A)** Morphological analysis of *P. bournei* under different PEG treatment times. The seedling was taken under the PEG treatment at 0, 1, 24, and 72 h. **(B)** Photosynthesis changes in *P. bournei* in response to PEG. **(C)** Oxidoreductase activity changes in *P. bournei* in response to PEG. **(D)** ABA content changes in *P. bournei* in response to PEG. Data are means and standard deviation calculated from three independent experiments. Different lowercase letters represent significant differences (*P*<0.05).

To better present the change in roots, hydroponic culture with PEG was employed for stimulated drought stress treatment. PEG-induced stress affected normal plant growth with browning roots (**Figure 1A**). The color gradually deepened with increasing treatment time. However, the leaves did not change significantly during the treatment.

Photosynthesis is an important indicator for evaluating the growth status of plants. Compared with the control, the photosynthetic parameters of *P. bournei* decreased to varying degrees after drought stress (**Figure 1B**). With increasing treatment time, the net photosynthetic and transpiration rates of *P. bournei* gradually decreased and were only 26.72% and 49.28%, respectively, of those of the control group after 72 h of treatment. The net photosynthetic and transpiration rates presented significant differences at each time point. The stomatal conductance of *P. bournei* leaves under stress conditions was not significantly different from that of the control at 1 h, while it decreased to 51.46% of that of the control at 72 h. The photochemical efficiency of PSII in the light (Fv/Fm) decreased significantly immediately after stress induction (1 h). However, there was no significant correlation between the stress duration and the Fv/Fm value.

Dehydration induces oxidative stress in plant cells, while plants themselves produce antioxidants such as POD and SOD to mitigate this damage. The changes in POD and SOD in *P. bournei* presented a similar pattern (**Figure 1C**). In the leaves, there were no significant differences in the POD and SOD activities between the control and treated groups after 1 h. However, the POD and SOD activities of the treated groups were significantly higher than those of the control group at 24 h and 72 h. The highest activities were observed at 24 h, with 52.22% and 42.46% increases in POD and SOD activities, respectively, compared to the control group. In the roots, the activities of POD and SOD gradually increased with treatment time, and the antioxidant enzyme activity was highest at 72 h.

Since ABA is a significant phytohormone that regulates the plant stress response, we monitored the levels of ABA in the leaves and roots of the stressed plants (**Figure 1D**). The ABA content in leaves gradually increased with increasing treatment time. The ABA concentration was much lower in roots than in leaves. The change in the ABA content in roots lagged behind that in leaves and significantly differed between the control and treated groups at 72 h.

### Transcriptomic changes in *P. bournei* under PEG treatments

To investigate the molecular response of *P. bournei* to drought stress, we conducted a comparative analysis of transcriptomic changes in the leaves and roots under three time points. After rRNA and low-quality reads were filtered out, the clean reads ranged from 36.73 to 54.81 Mb across the libraries, with 71.06 to 89.53% of sequenced reads being accurately mapped to the *P. bournei* genome (**Supplementary Table 2**).

**Figure 2:**
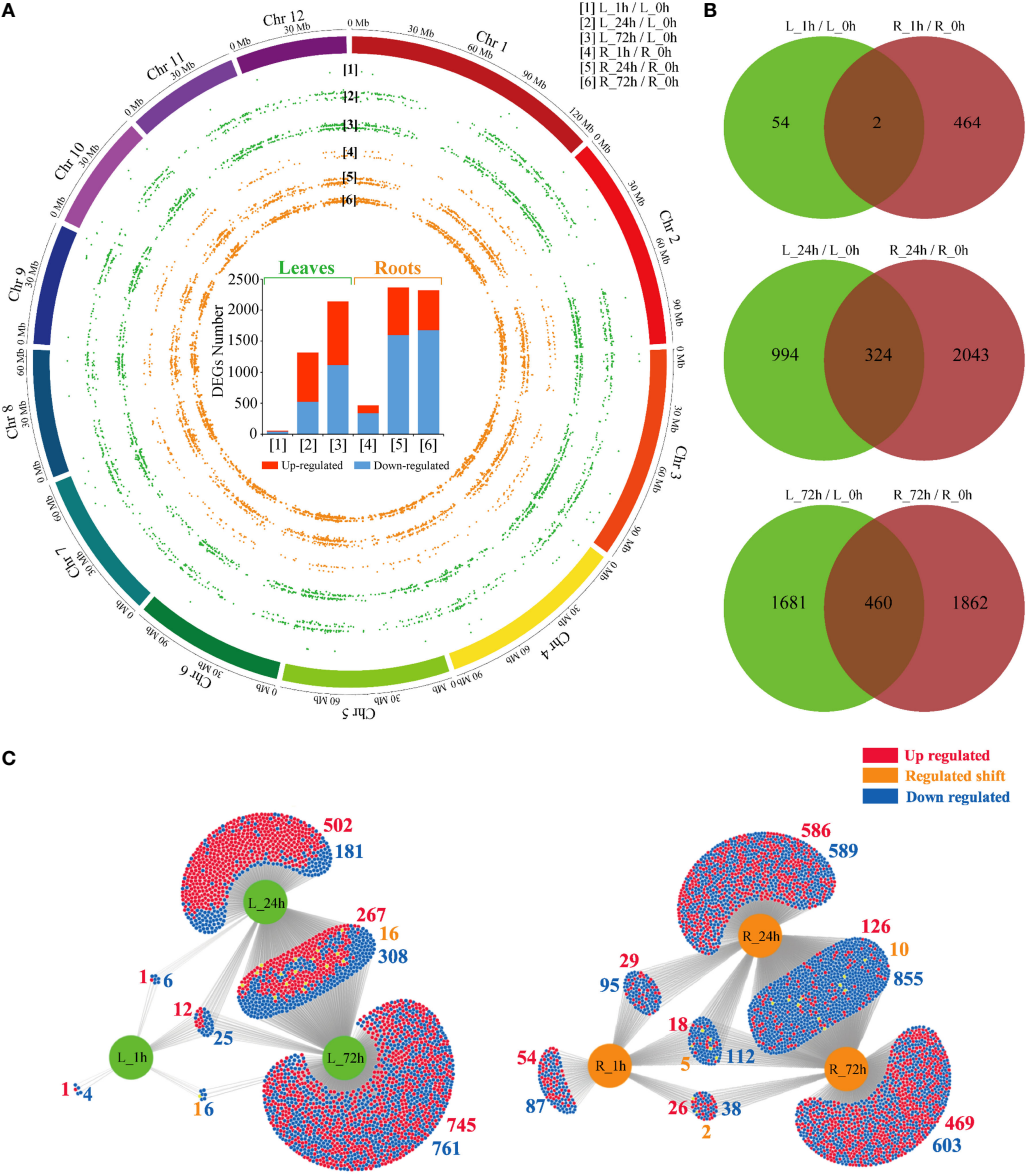
Overview of PEG treatment effects on *P. bournei* gene expression. **(A)** Statistical histogram of the DEGs and distribution in 12 P*. bournei* chromosomes from pairwise comparisons. **(B)** Venn diagram analysis of DEGs among different treatment periods between leaves and roots. **(C)** DiVenn diagram analysis of DEGs among different treatment periods in leaves and roots.

A total of 5688 DEGs (**Supplementary Table 3**) were identified, and the distribution analysis across the *P. bournei* chromosomes indicated that they were not significantly enriched in any specific chromosome region (**Figure 2A**). The number of DEGs in *P. bournei* was significantly positively correlated with stress duration. Significantly fewer DEGs were identified in the leaves (58, 1318, and 2141) than in the roots (466, 2376, and 2322) at the respective time points of 1 h, 24 h, and 72 h, indicating a slower stress response rate in leaves. The stress-responsive transcriptomic program was predominant in the roots.

We then compared the DEGs overlapping among different stress stages between *P. bournei* leaves and roots (**Figure 2B**). The results showed that the stress-responsive genes in the leaves and roots presented independent expression profiles. Only two genes, *Kiss Me Deadly 3* (*KMD3*), which controls phenylpropanoid biosynthesis, and *Arabidopsis Toxicos En Levadura 16* (*ATL16*), which encodes a RING/U-box superfamily protein, exhibited differential expression in both tissues at 1 h. As the duration of stress increased, the number of genes that acted in unison in leaves and roots also gradually increased. Functional annotation analysis revealed that the overlapping DEGs between leaves and roots were mainly involved in biological processes such as protein phosphorylation, oxidation-reduction processes, and carbohydrate metabolic processes.

The overlaps in each comparison were shown in a DiVenn diagram to identify identical DEGs in different stress stages (**Figure 2C**). The number of up-regulated and downregulated DEGs in the overlap of L_24h and L_72h was almost the same. A total of 502 up-regulated genes were enriched in L_24h. In contrast, the overlap of the DEGs in the roots was mainly downregulated. The highest overlap of the DEGs was between R_24h and R_72h (991). There were 855 downregulated genes, 126 up-regulated genes, and 10 genes with varying expression patterns. The overlap of the three conditions consisted of 135 DEGs in roots and 37 DEGs in leaves. Compared with leaves, there were more genes with changed expression trends in the three overlapping conditions of the roots. This result suggests that the responsive genes at different stress times are unique.

### Gene ontology enrichment in DEGs

**Figure 3:**
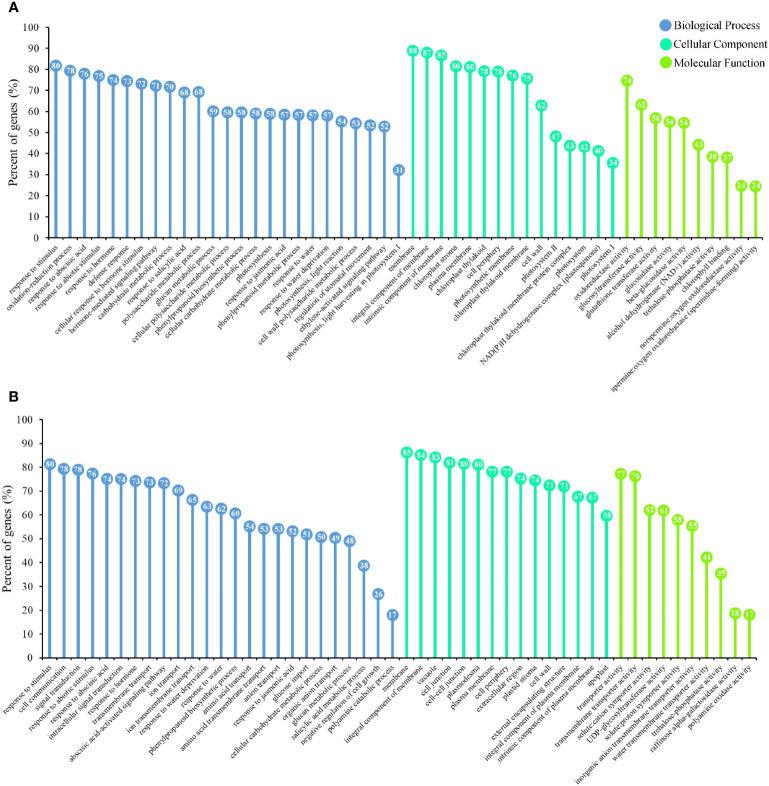
*P. bournei* DEGs functional classification by gene ontology (GO) analysis. **(A)** Enrichment lollipop diagram of GO terms assigned to DEGs in leaves. **(B)** Enrichment lollipop diagram of GO terms assigned to DEGs in roots.

GO was utilized as a universal functional classification system to identify the primary molecular functions of the DEGs. The DEGs of leaves and roots were used separately for GO analysis and screen representative terms (**Figure 3**). We found that the DEGs were enriched in many biological processes associated with the drought-stress response. The response to stimulus (GO:0050896) was the most significantly enriched biological process (BP) term in the DEGs in both leaves and roots. The BP of DEGs in leaves mainly included photosynthesis (GO:0015979, GO:0019684, GO:0010119, and GO:0009768), the hormonal response (GO:0009737, GO:0009725, GO:0032870, GO:0009755, GO:0009751, GO:0009753, and GO:0010105) and carbohydrate metabolism (GO:0044262, GO:0044264, GO:0044042, GO:0044264, GO:0044262, and GO:0044264) (**Figure 3A**). Cellular components (CC), such as the chloroplast stroma (GO:0009570), photosynthetic membrane (GO:0034357), and photosystem (GO:0009521), were enriched in the DEGs in leaves. The DEGs in leaves were primarily involved in molecular functions (MFs) closely related to the drought stress response, such as oxidoreductase activity (GO:0016491), glutathione transferase activity (GO:0004364), and trehalose-phosphatase activity (GO:0004805). Unlike the GO-enriched terms in leaves, the DEGs in roots were mainly related to transport and signal transduction (**Figure 3B**). The terms cell communication (GO:0007154), signal transduction (GO:0007165), anion transport (GO:0006820), and ion transport (GO:0006811) all had a relatively high degree of enrichment. Among the CC of DEGs in roots, six terms were related to the membrane (GO:0005886, GO:0016020, GO:0016021, GO:0031226, GO:0009532, and GO:0005887), and three terms related to cell junctions (GO:0009506, GO:0005911, and GO:0030054) were highly enriched. In addition to the activities of carbohydrate synthase (GO:0008194, GO:0004805, and GO:0052692) related to drought stress, many MFs related to transporter activities were also enriched in the DEGs in roots.

### Transcription factor identification

**Figure 4:**
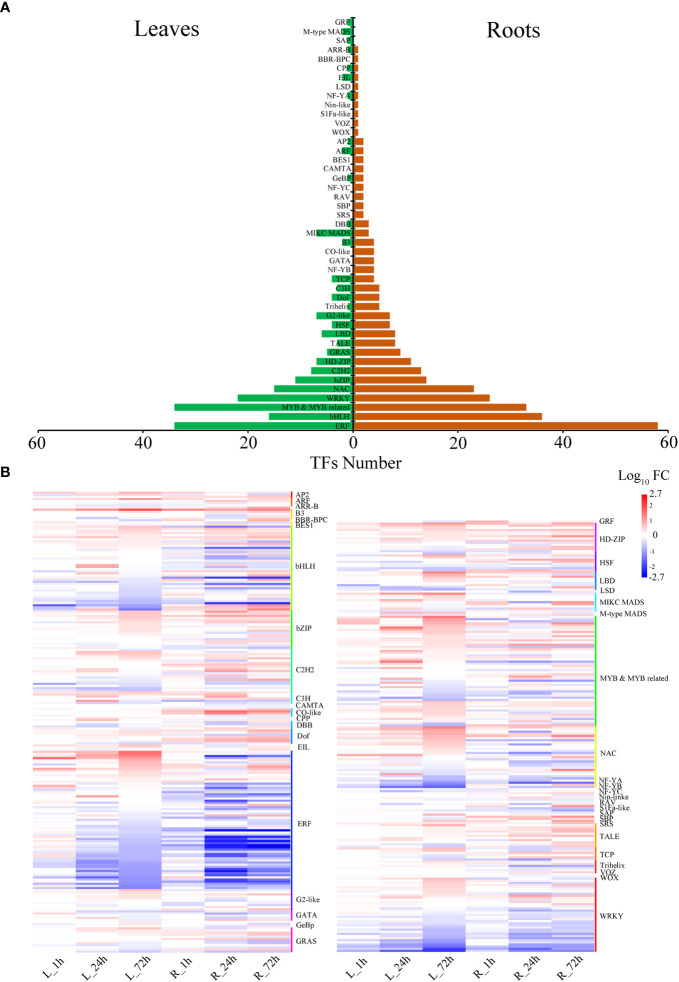
Distribution of transcription factors responsive to drought stress in *P. bournei*. **(A)** The number of predicted TFs in the leaves and roots. Each row in the figure corresponds to a TF gene family. The green represents the number of TFs in the leaves, and the brown represents the number of TFs in the roots. **(B)** Heatmap of the expression of all TFs at different treatment periods in the TF family with cluster assignments shown on the right. Heatmap indicates log 10 (fold change) of genes where red indicates up-regulated and blue indicates down-regulated genes.

Drought stress significantly affected the expression of 447 TFs. The MYB, ERF, WRKY, bHLH, and NAC genes accounted for a significant proportion of *P. bournei* (**Figure 4**). In leaves, there were 207 TFs in 30 gene families, with MYB exhibiting the highest number of genes (**Figure 4A**). Compared to leaves, a greater diversity of transcription factor families was found in roots, with ERF exhibiting the highest number of genes. The BBR-BPC (Phbou.02G0563), CO-like (Phbou.05G2008, Phbou.03G2012, and Phbou.10G1237), SBP (Phbou.05G1740 and Phbou.05G1326) and SRS (Phbou.02G2683) family members were explicitly up-regulated in roots. The GRF (Phbou.01G2410), M-type MADS (Phbou.09G1428), and SAP (Phbou.03G1232) family members were explicitly up-regulated in leaves (**Figure 4B**). Every gene family of TFs had variable expression patterns in the different treatment periods and organs. Some exceptions, such as the B3 (Phbou.01G2687), C3H (Phbou.03G3182), HD-ZIP (Phbou.07G1018) and NAC (Phbou.07G0803, Phbou.03G0565) family members, had the same up-regulated pattern during all three treatment periods in both leaves and roots, and the TPM levels all exceeded twice those of the 0 h.

### Weighted gene co-expression network analysis module generation and core TF analysis

**Figure 5:**
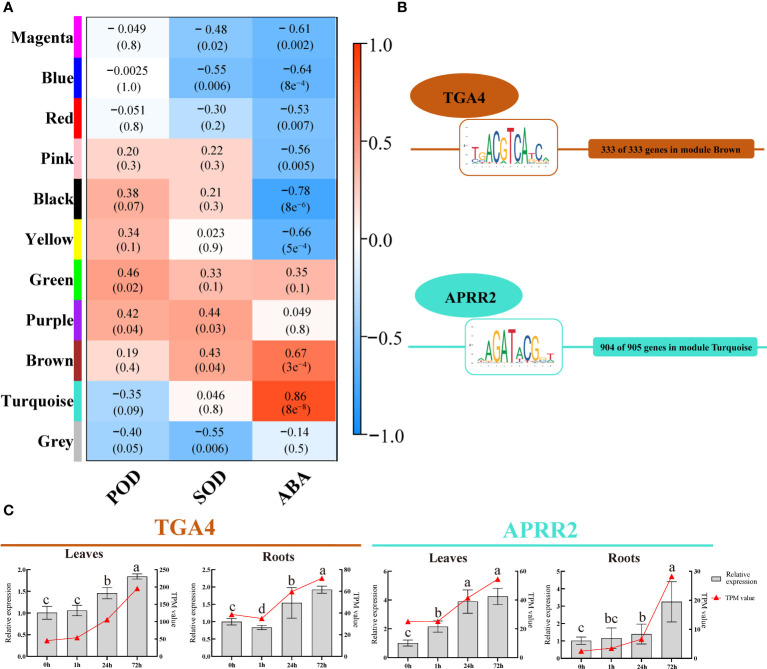
WGCNA and core TFs and their binding sites (TFBS) identification. **(A)** Module-Trait correlations. Each row in the table corresponds to a module and each column to a physiological index. The module name is shown on the left side of each cell. Numbers in the table represent the correlations between the corresponding module eigengenes and physiological indices, with p values listed below the correlations in parentheses. The table is color-coded to represent correlation strength according to the color legend. The intensity and direction of each correlation are indicated on the right side of the heatmap (The red indicates a positive correlation, and the blue indicates a negative correlation). **(B)** The most abundant TFBS and its corresponding TF in brown and turquoise modules. **(C)** Expression validation of the *PbTGA4* and *PbAPRR2* using qRT-PCR in 4 periods of leave and roots. Data are means and standard deviation calculated from three independent experiments. Different lowercase letters represent significant differences (*P*<0.05).

We utilized WGCNA to analyze the gene co-expression patterns of *P. bournei* in response to drought to investigate the correlation between genes and physiological indices, as well as intramodular and intermodular genes. A total of 4584 DEGs (TPM>1) were analyzed, and 11 co-expression modules and correlation coefficients were identified and obtained (**Figure 5A**). The turquoise module was positively correlated with ABA, and the correlation coefficient was 0.86. The brown module has a significant positive correlation with SOD and ABA. Therefore, the two modules were selected to screen the hub genes and analyze the network.

For each module, TFs in this module were selected as prior regulators. After transcription factor-binding site (TFBS) scanning, 21 TFs belonging to 13 TF families had TFBSs of genes in the brown module, and 24 TFs belonging to 14 TF families had TFBSs of genes in the turquoise module. A total of 333 genes in the brown module all had TFBSs belonging to *TGACG Motif-Binding Factor 4* (*PbTGA4*) of the bZIP family, and 904 out of 905 genes in the turquoise module had TFBSs belonging to *Arabidopsis Pseudo-Response Regulator 2* (*PbAPRR2*) of the ARR-B family. These two TFs were selected as core TFs in each module (**Figure 5B**, **Supplementary Table 4**).

The relative expression of two core TFs was strongly induced by drought stress in plants (**Figure 5C**). The results of transcriptomic sequencing and qRT-PCR showed the same trend. This result further verified the model of the hub gene in response to drought and the reliability of the transcriptomic data.

### Core TF co-expression network analysis and functional enrichment analysis

**Figure 6:**
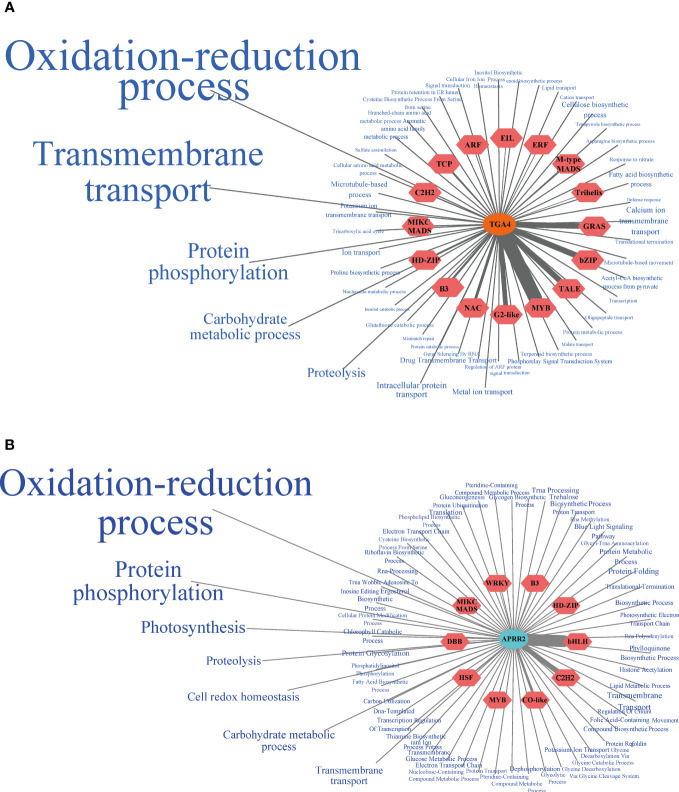
Core TFs co-expression network. **(A)** *PbTGA4* co-expression network of the brown module. **(B)** *PbAPARR2* co-expression network of the turquoise module. The TFs co-expressing with the core TF were located in the red hexagon, and the thickness of the line connection between them reflects the weight value. The blue font on the periphery was the biological process that co-expresses functional genes with core TF, and the font size reflects the weight value of the genes contained in each GO term and core TF.

Plants’ response to drought stress is a collaborative process. Core TFs and their interconnected TFs and functional genes should be considered when regulating the plant response to drought. Based on two core TFs screened by WGCNA, we proposed a regulatory network of hub and edge genes closely related to them. In the brown module, *TGA4* showed a complex co-expression relationship with 330 genes in the module (**Supplementary Table 5**). Thirty-five genes from 17 TF families, such as the HD-ZIP and MIKC MADS families, showed a high co-expression relationship with *PbTGA4*. The functional genes co-expressed with *PbTGA4* mainly participated in the oxidation-reduction process (GO:0055114), transmembrane transport (GO:0055085), protein phosphorylation (GO:0006468), carbohydrate metabolic process (GO:0005975) and proteolysis (GO:0006508) (**Figure 6A**), such as *cytochrome P450-71* (*CYP71*, Phbou.010C003), *CYP76* (Phbou.08G1384), *CYP97* (Phbou.07G1158), *CYP707* (Phbou.09G1221), *aldehyde dehydrogenase 7B4* (*ALDH7B4*, Phbou.12G1648), *PIN-Formed 3* (*PIN3*, Phbou.10G1119), *polyol *& *monosaccharide transporter 1* (*PMT1*, Phbou.11G0533) and *open stomata 1* (*OST1*, Phbou.06G2001). In the turquoise module, *PbAPRR2* exhibited a high relationship with 380 edge genes. The TFs with a high weight value came from the bHLH family, C2H2 family, and WRKY family (**Figure 6B**, **Supplementary Table 6**). The genes co-expressed with *PbAPRR2* were mainly involved in the oxidation-reduction process (GO:0055114), protein phosphorylation (GO:0006468), photosynthesis (GO:0015979), proteolysis (GO:0006508) and cell redox homeostasis (GO:0045454), such as *CYP706* (Phbou.02G0977), *glutamate synthase 1* (*GLU1*, Phbou.12G1524), cysteine-rich *receptor-like protein kinase 10* (*CRK10*, Phbou.07G0003), *thioredoxin M-type 4* (*TRX-M4*, Phbou.02G3206) and *photosynthetic NDH subcomplex B4* (*PNSB4*, Phbou.03G3345). By conducting qPCR experiments on the high co-expression levels of transcription factors, we discovered that these genes demonstrated comparable expression patterns under stress conditions in both tissues (**Supplementary Figure 1**). This finding strengthens the credibility of the transcriptome sequencing outcomes.

## Discussion

Distinguishable from the short life cycle of annual herbaceous plants to escape stress, perennial woody plants suffer prolonged and repeated stresses. The growth and biomass accumulation of woody plants is inhibited under water shortage, which becomes more evident with increased stress duration (Zlobin, 2022). Thus, trees evolve complex regulatory networks to adjust their growth patterns and biomass allocation for adaptation to water-limited environments (Jia et al., 2022). This study combined morphological, physiological, and transcriptomic changes to identify genes differentially expressed under a drought-mimicking treatment. The regulatory networks of *P. bournei* in response to PEG treatments were initially elucidated.

### The inconsistent responses of morphological and physiological changes in *P. bournei* under short-term drought stress

Plants undergo physiological and morphological changes during drought stress (Fang et al., 2022). Extensive research has shown that drought stress increases ABA accumulation in leaves to induce stomatal closure and reduce water loss (Yu et al., 2021). Photosynthesis is impaired due to stomatal closure and limited carbon dioxide intake (Salomon et al., 2022). Drought induction significantly reduced the net photosynthetic rate, stomatal conductance, and transpiration rate in *P. bournei*. At the same time, GO enrichment analysis suggested an enrichment of photosynthesis-related terms in the leaves under drought stress induction. The results are consistent with previous conclusions (Razi and Muneer, 2021). The Fv/Fm ratio reflects the function of the whole PSII, and its reduction indicates the damage or photoinhibition of PSII in response to environmental stress (Yan K. et al., 2022). *P. bournei* had a significantly lower Fv/Fm value than the control at 1 h of drought induction, indicating that 10% PEG caused drought stress.

Drought stress causes lipid peroxidation and cell membrane damage due to ROS accumulation. Plants prevent cellular damage from ROS accumulation by increasing the activities of SOD and POD (Yan W. P. et al., 2022). However, severe drought can lead to a decrease in the efficiency of the antioxidant defense system in plants, decreasing the activities of antioxidant enzymes such as SOD and POD (Chen et al., 2017). The SOD and POD activities of *P. bournei* leaves initially increased and then decreased in response to drought induction. At the same time, the activity of SOD in roots showed a positive correlation with the duration of drought stress. Furthermore, the activity of POD in roots was higher than in leaves. These results indicate that the initial increase in SOD and POD activities in leaves helps to resist drought-induced damage. However, prolonged drought conditions lead to a decrease in SOD and POD activities due to the decline in the efficiency of the antioxidant defense system.

ABA plays an essential role in the response to drought stress in plants (Xue et al., 2018). ABA alleviates the damage of drought stress caused to plants by closing stomata, mediating root architecture, and regulating stress-responsive gene expression (Gupta et al., 2020; Muhammad Aslam et al., 2022). In this study, the content of ABA in leaves was negatively correlated with photosynthesis, and the accumulation of ABA might be the main reason for the decrease in the stomatal conductance, transpiration rate, and net photosynthetic rate of *P. bournei.* The response to abscisic acid (GO:0009737), the cellular response to hormone stimulus (GO:0032870), and photosynthesis (GO:0015979) were enriched in leaves. A previous study showed that ABA is rapidly synthesized in leaves after plants are subjected to drought stress (Zhang F. P. et al., 2018). ABA synthesized in the leaves can be transported through the phloem to the roots, then enters the xylem and returns to the shoot (Yoshida et al., 2019). The ABA content in *P. bournei* roots exhibited a similar variation pattern to the above results. The biological process of response to abscisic acid (GO:0009737) was also enriched in roots. However, in contrast to leaves, signal transduction (GO:0007165) exhibits the highest enrichment in roots. We observed a significant increase in the expression level of genes in signal transduction, such as *sucrose non-fermenting 1-related protein kinase 3* (*SnRK3*, Phbou.03G0313), after 1 hour of treatment. *SnRK3* has a role as a component of calcium signaling, mediating plant development, and regulating responses to abiotic stress (Zhang et al., 2020). Therefore, we consider the genes involved in signal transduction to be crucial factors in coordinating the response of both aboveground and belowground plant parts to drought stress.

Unlike the solid physiological and molecular responses to drought stress, the leaves of *P. bournei* showed no significant morphological changes during the treatment period. Previous research showed different morphological responses to drought stress between leaves of evergreen and deciduous species (De Oliveira et al., 2015). To reduce the negative effects of drought stress and diminish variations in water potential, evergreen species adopt a water-use strategy that is highly conservative to maintain leaf shape (De Souza et al., 2020). In comparison, deciduous species tend to prioritize their water potential by facilitating leaf wilting and detachment to ensure their survival during a drought period (Lima et al., 2018). *P. bournei* is a typical evergreen species with an epicuticular waxy layer covering leaves that enhances drought stress tolerance (Al-Abdallat et al., 2014; Han et al., 2022). Transcriptomic analysis showed that the response speed of the differential expression of genes in leaves was slower than that in roots. In summary, the lack of significant morphological changes in the leaves of *P. bournei* during drought treatment might be attributed to the characteristics of evergreen tree species, such as the wax layer of leaves and the relatively slow response of gene expression. Due to this characteristic, it may not be possible to determine promptly whether *P. bournei* experienced drought stress only by observing changes in the aboveground parts during seedling cultivation and afforestation.

### ABA-related core TFs that were differentially regulated in response to drought stress

To harvest the potential biological significance from the abundant DEGs, categorizing these genes into modules using co-expression profiling could help us better identify key genes involved in the response to drought stress. We identified two core TFs, *PbTGA4* and *PbAPRR2*, from the ABA-related brown and turquoise modules, respectively. *PbTGA4* exhibited a co-expression relationship with many CYP family member genes involved in the oxidation-reduction process and *ALDH7B4* in the co-expression network. *TGA* transcription factors belong to the D subfamily of the basic region-leucine zipper (bZIP) family. As a TF directly regulated by ABA, *TGA* plays a significant role in various abiotic stresses and signal transduction pathways (Sakuraba et al., 2015). *TGA* transcription factor family members regulate gene expression and participate in the plant stress response by binding to TGACG motifs on DNA promoters (Ju et al., 2012; Van Der Does et al., 2013). In addition, they can also interact with signaling transduction proteins in the ABA and jasmonic acid (JA) pathways and regulate their activity, thus participating in plant resistance to drought stress (Zander et al., 2010). Cytochrome P450 is an important class of enzymes present in plants. Under drought stress, cytochrome P450 may regulate the synthesis and decomposition of antioxidants in plant cells, thus regulating the oxidative/reductive state in cells to reduce ROS accumulation and promote plant adaptation to drought stress (Zhou et al., 2023). Plants will increase the expression level of *ALDH* to prevent the accumulation of aldehyde substances that may cause damage in plant cells after being subjected to abiotic stress (Hou and Bartels, 2015). This result suggested that *PbTGA4* might regulate genes with oxidation-reduction process functions to alleviate ROS accumulation and the toxicity of aldehyde substances caused by drought stress in *P. bournei*.

*APRR2* is a transcription factor involved in phytochrome synthesis (Yang et al., 2015). Apart from co-expression with the CYP family in *P. bournei* under drought stress, it was observed in this study that *PbAPRR2* also showed a significant degree of co-expression with amino acid synthesis and protein kinase-related functional genes such as *GLU1*, *CRK10*, and *TRX-M4*. *MdAPR2* promoted chlorophyll synthesis by activating the expression of chlorophyll synthesis-related genes to alleviate stress damage to apple (*Malus domestica*) leaves (Wen et al., 2022). *APRR2* has been reported to regulate the synthesis of flavonoids and antioxidant enzymes, which can alleviate oxidative stress induced by drought stress in plants (Perochon et al., 2010). Additionally, *APRR2* participates in the ABA-dependent response to abiotic stress by interacting with transcription factors (Kurup et al., 2000; Wen et al., 2022). This result indicated that *PbAPRR2* carries out a complex regulatory function in the response of plants to drought stress.

### Potential molecular mechanism of the response to drought stress in *P. bournei*

**Figure 7:**
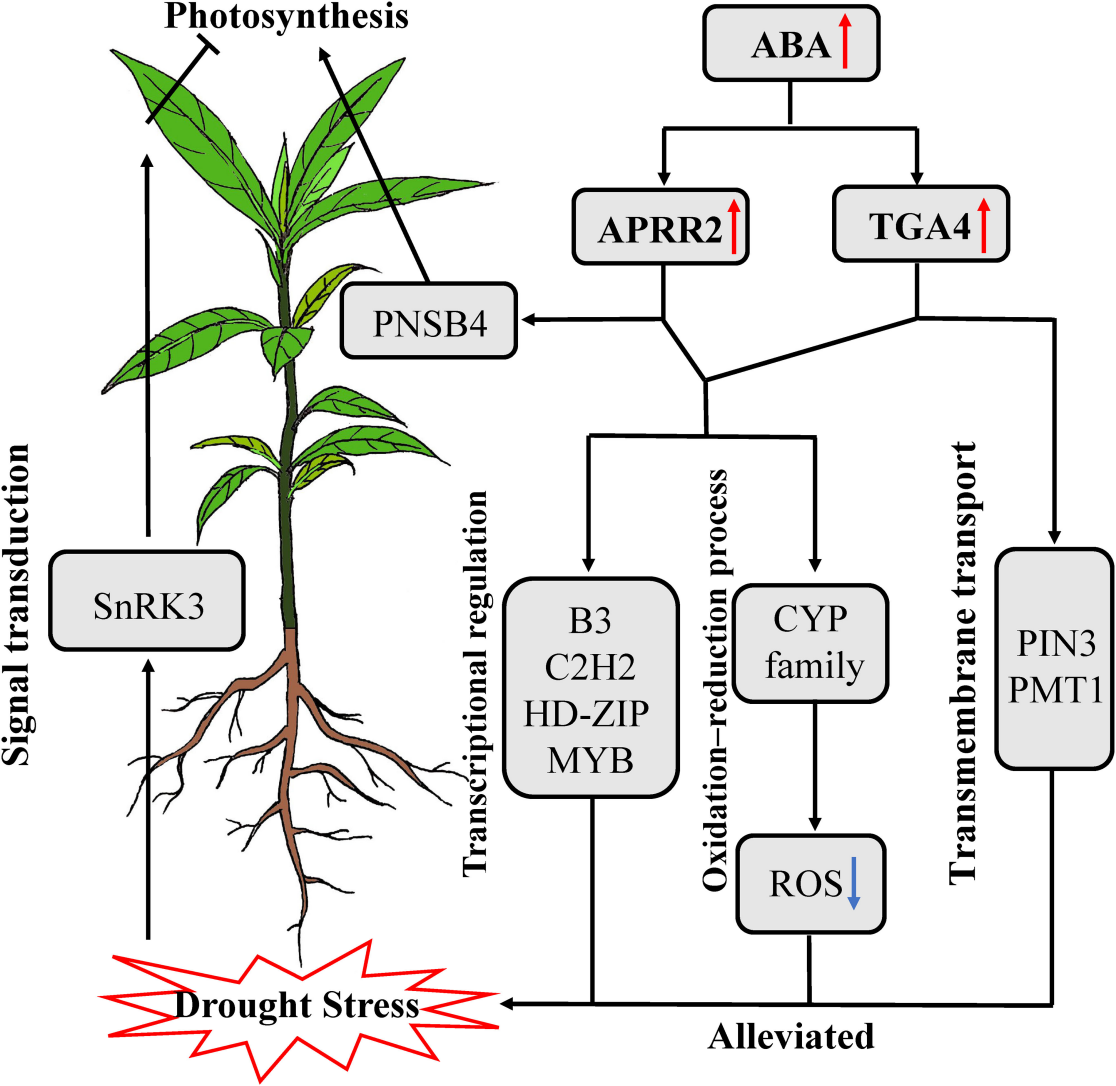
The model for the response to short-term drought stress in *P. bournei*.

Based on current research, we proposed a model to explain how *P. bournei* alleviates drought stress (**Figure 7**). When *P. bournei* roots sense water deficiency, they transmit the stress signal to aboveground tissues by activating signal transduction genes such as *SnRK3*. Upon receiving the signal, *P. bournei* leaves synthesize ABA to regulate stomatal closure to maintain water potential, but this inhibits photosynthesis and causes the accumulation of ROS. ABA accumulation activates the downstream transcription factors *PbAPRR2* and *PbTGA4*. *PbAPRR2* and *PbTGA4* alleviate ROS damage to cells by regulating genes involved in oxidation-reduction processes in *P. bournei*. The upregulation of core TFs influences other TFs and functional genes, inducing physiological and biochemical changes in *P. bournei* to enhance drought stress tolerance. However, in the plant response to drought stress, *PbAPRR2* correlated more with photosynthesis genes, while *PbTGA4* was more inclined to be co-expressed with transmembrane transport genes. This result indicated that *PbAPRR2* and *PbTGA4* also involve different response pathways to help *P. bournei* resist drought stress.

## Data availability statement

The datasets presented in this study can be found in online repositories. The names of the repository/repositories and accession number(s) can be found in the article/**Supplementary Material**.

## Author contributions

JY: Data curation, Investigation, Methodology, Writing – original draft. KY: Data curation, Investigation, Methodology, Writing – original draft. YL: Investigation, Methodology, Writing – original draft. YHL: Investigation, Methodology, Writing – original draft. JZ: Investigation, Writing – review & editing. XH: Data curation, Supervision, Writing – review & editing. ZT: Project administration, Supervision, Writing – review & editing.
