# Supplementary figures and images for: Co-expression network analysis reveals PbTGA4 and PbAPRR2 as core transcription factors of drought response in an important timber species Phoebe bournei
Source: Front Plant Sci. 2024 Jan 8;14:1297235. doi: 10.3389/fpls.2023.1297235 (PMC10800493; doi:10.3389/fpls.2023.1297235)

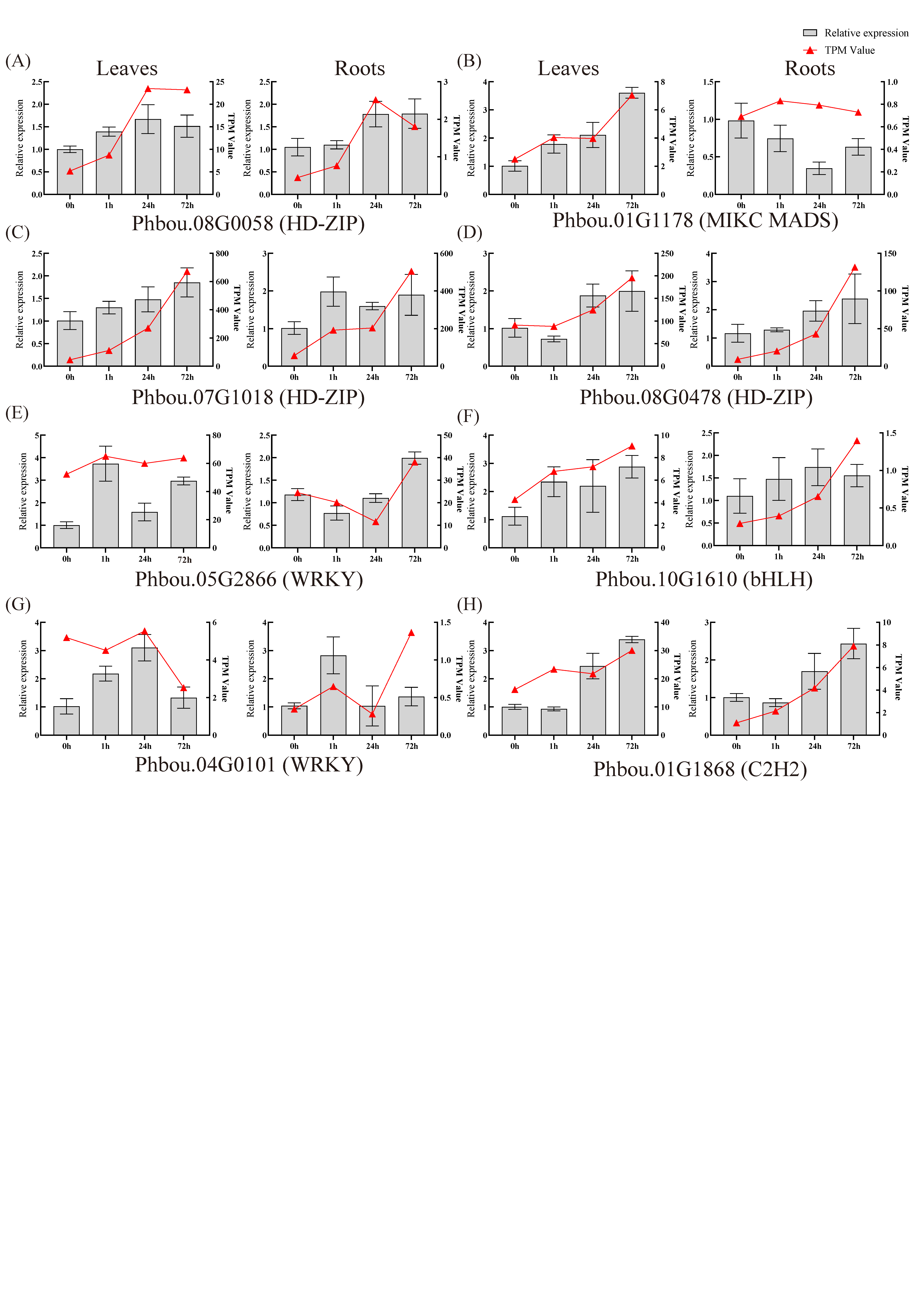

Supplement: Supplementary file 1 [file Image_1.tif]
